# Localized *in-situ* deposition: a new dimension to control in fabricating surface micro/nano structures via ultrafast laser ablation

**DOI:** 10.1007/s12200-023-00092-1

**Published:** 2023-11-17

**Authors:** Peixun Fan, Guochen Jiang, Xinyu Hu, Lizhong Wang, Hongjun Zhang, Minlin Zhong

**Affiliations:** Laser Materials Processing Research Centre, Key Laboratory for Advanced Materials Processing Technology (Ministry of Education), School of Materials Science and Engineering, Tsinghua University, Beijing, 100084 China

**Keywords:** Ultrafast laser ablation, Laser micro/nanofabrication, Surface micro/nano structures, *In-situ* deposition, Micro-additive fabrication

## Abstract

Controllable fabrication of surface micro/nano structures is the key to realizing surface functionalization for various applications. As a versatile approach, ultrafast laser ablation has been widely studied for surface micro/nano structuring. Increasing research efforts in this field have been devoted to gaining more control over the fabrication processes to meet the increasing need for creation of complex structures. In this paper, we focus on the *in-situ* deposition process following the plasma formation under ultrafast laser ablation. From an overview perspective, we firstly summarize the different roles that plasma plumes, from pulsed laser ablation of solids, play in different laser processing approaches. Then, the distinctive *in-situ* deposition process within surface micro/nano structuring is highlighted. Our experimental work demonstrated that the *in-situ* deposition during ultrafast laser surface structuring can be controlled as a localized micro-additive process to pile up secondary ordered structures, through which a unique kind of hierarchical structure with fort-like bodies sitting on top of micro cone arrays were fabricated as a showcase. The revealed laser-matter interaction mechanism can be inspiring for the development of new ultrafast laser fabrication approaches, adding a new dimension and more flexibility in controlling the fabrication of functional surface micro/nano structures.

**Graphical Abstract:**

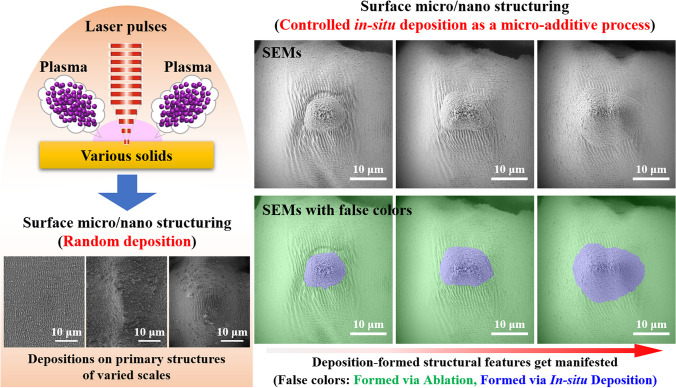

## Introduction

Surface functionalization of various materials has great importance for a variety of applications. The preparation of micro/nano structures in a controlled fashion is essential for surface functionalization with tunable properties, and is driving the continuous progress of micro/nano fabrication techniques [1–5]. In particular, ultrafast lasers have been proven to be efficient and versatile tools for fabricating diverse surface micro/nano structures at both single and multiple scales, possessing distinctive advantages of maskless, non-contact, low heat effect, material independence, and high flexibility, etc*.* [5–10]. The physical processes involved in ultrafast laser-matter interaction are complicated. Through ultrafast laser ablation, in particular, laser-inscribed and laser-induced surface structures have been produced. Regular and arrayed microscale structures are mainly fabricated via the inscribing process, with sub-microscale features being induced simultaneously [11, 12]. The inscribing process is a typical subtractive approach where the structure sizes and profiles are mainly determined by the laser beam qualities and laser scanning strategies. Nano ripples (or laser induced periodic surface structures, LIPSS) are the most representative and unique laser-induced features and have been extensively studied in the past decade [13–16]. To date, high controllability has been achieved within both the inscribing and inducing processes, facilitating the fabrication of plentiful surface structures with freely designed patterns, orientations, and morphologies.

**Fig. 1:**
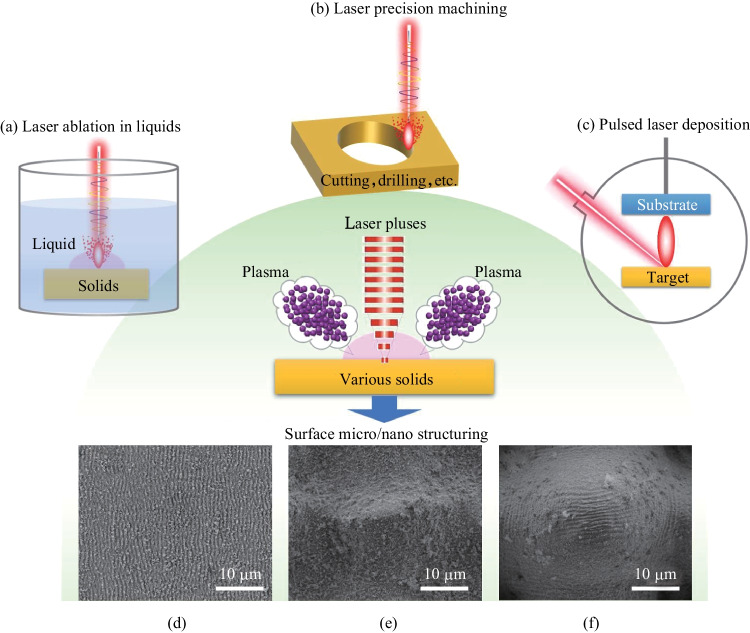
Overview of the universal plasma-plume formation process during pulsed laser ablation of solids with different laser processing approaches (**a** laser ablation in liquids, **b** laser precision machining, and **c** pulsed laser deposition) and the varied outcomes after the plasma formation. **d**–**f** show the re-deposited particle features randomly distributed on primary surface structures, of different scales, inscribed and induced by ultrafast laser. As can be seen, the re-deposition of energetic species during ultrafast laser surface micro/nano structuring is an *in-situ* process, distinctive from the approaches in (**a** − **c**)

Another key factor influencing the surface micro/nano structures produced by ultrafast lasers is the formation of plasma plumes composed of energetic species including atoms, clusters, particulates, etc., and their re-deposition on irradiated surfaces to form particle features varying in scale from tens of nanometers to several microns. From a broader view, the plasma plume formation is a universal phenomenon during pulsed laser ablation of solids. It is a phenomenon that plays different roles within different laser processing approaches, as illustrated in Fig. 1. In laser ablation in liquids, the energetic species in plasma plumes can be collected by the liquids and used for optoelectronic and catalytic applications [17, 18]. In pulsed laser deposition, the energetic species in plasma plumes are used to prepare functional thin films on off-target substrates (where film formation and laser ablation occur on separate surfaces) [19]. However, the plasma plumes can result in lower processing efficiencies through shielding or lower kerf qualities with debris, which should be avoided within high-precision laser machining [20, 21].

Unlike the energetic species being collected for usage by external liquids (in the case of laser ablation in liquids) or substrates (in the case of pulsed laser deposition), the energetic species during ultrafast laser surface micro/nano structuring experience re-deposition *in-situ* on the surfaces being processed. The *in-situ* deposition and formation of particle features is usually considered as an accompanying effect of both the laser inscribing and inducing processes (Fig. 1), with the *in-situ* deposited particles considered as decorative features on the primary structures [22, 23]. In spite of the progress in utilizing the *in-situ* deposition process for surface structuring during ultrafast laser ablation of various materials including metals [24, 25], silicon [26, 27], ceramics [28], glasses [29], and polymers [30], the process is usually random. The surface areas for *in-situ* deposition to occur cannot be designated and the secondary structures formed in this case are usually simple. Whether and how the *in-situ* deposition process can be controlled remains an open question. In recent years, as studies on surface functionalization and micro/nano structuring have flourished, the significances of the *in-situ* deposited structural features for enhancing various surface properties, e.g., light absorption [25, 26, 31], sensibility [24, 32], and energy conversion [33], have been identified. There have thus been increasing efforts to gain a better control over the *in-situ* deposition process [31–35].

In this work, we report that the *in-situ* deposition during ultrafast laser ablation can be controlled as a localized micro-additive process, piling up ordered structures rather than randomly distributed particles. We show that both the laser power and scanning strategies determine the *in-situ* deposition based micro-additive process. Tungsten was selected as the showcase material considering its high melting point and high hardness, which could benefit the control over the micro/nano structure formation. Though carefully controlling the ultrafast laser conditions, alternate and sequential subtractive (ablation) and additive (*in-situ* deposition) processes could be implemented. As a specific demonstration, a unique kind of micro/nano hierarchical structures with ordered micro cone arrays capped by fort-like structures with controlled sizes, built of the *in-situ* deposited nanoparticles, were fabricated. The controlled *in-situ* deposition can add a new dimension and more flexibility in controlling the fabrication of surface micro/nano structures using ultrafast lasers.

## Experiment

The experiments employed a Trumpf TruMicro 5000 femtosecond (fs) laser system, generating 800 fs pulses at a central wavelength of 1030 nm, for the surface micro/nano structure fabrication. Before laser processing, the tungsten plates (99.9% purity) were mechanically polished to mirror finish and cleaned ultrasonically with ethanol to remove oxide and grease from their surfaces. An *x*–*y* galvanometer scanner was used to focus and scan the laser beam on sample surfaces in a pattern of cross lines in atmospheric environment. The diameter of the focused spot, defined by an intensity drop to 1/e^2^ of the maximum value, was approximately 35 μm. Different laser processing parameters including laser power, pulse repetition rates, scanning speeds, intervals of scanning lines (i.e., periodicities of arrayed microscale structures), and repeated number of laser scans were systematically investigated. The evolution of surface micro/nano structures with various laser processing parameters was carefully characterized using a field emission scanning electron microscope (SEM).

## Results and discussion

**Fig. 2:**
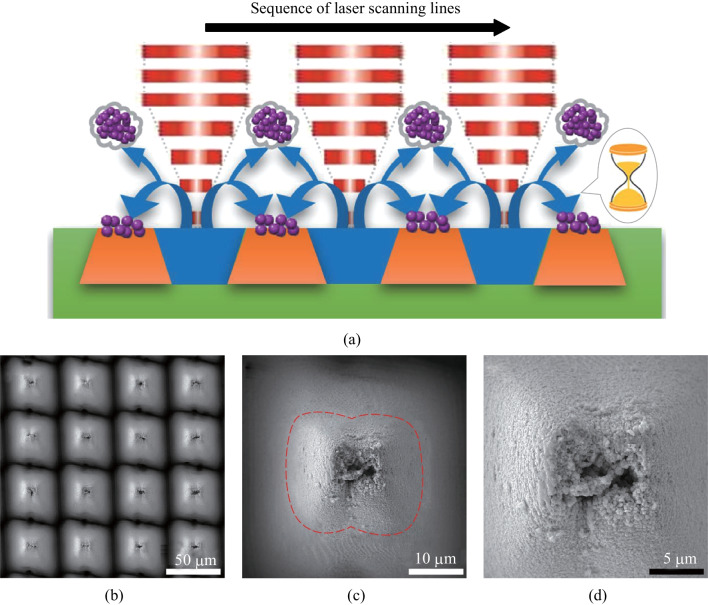
**a** Schematic illustration of the sequential subtractive and additive fabrication processes under femtosecond laser irradiation (Green-Tungsten plate; Blue-Ablated area; Orange-Unablated area. The black arrow shows the sequence of laser scanning lines). **b**–**d** SEM images in different magnifications of a particular kind of hierarchical structures prepared via the femtosecond laser ablation and *in-situ* deposition process. **b** Micro cone arrays with a periodicity of 50 μm formed by the subtractive ablation process. **c** Secondary structures (marked within the red dash rectangle) on top of the micro cone arrays formed by the additive *in-situ* deposition process. **d** Sub-micro features constituting the secondary structures

Previous work has shown that during high intensity femtosecond laser irradiation, the surface areas directly irradiated by laser pulses are ablated, and plasmas consisting of energetic species like atoms, clusters, and particulates are formed [36, 37]. These energetic species are ejected to the ambient atmosphere with high initial velocities, which is a typical subtractive process. Intense collisions of the energetic species occur during their dissipation, making them aggregate to form nanoscale particle features. Some of the energetic species as well as the nanoparticles formed can finally dissipate into the ambient atmosphere, while others fall back onto the solid surfaces due to gravity and the ambient pressure, especially onto the areas not being directly irradiated by laser pulses, as illustrated in Fig. 2a. The falling back process of the nanoparticles resembles the fall of sand in an hourglass, but occurs* in-situ* on the surfaces being ablated. Usually, the *in-situ* deposition results in randomly distributed nanoparticles covering the primary structures produced by laser (a typical example of the primary structures can be arrayed micro cones). During the femtosecond laser ablation of tungsten, however, we noticed that the *in-situ* deposition can be used as a localized micro-additive process to form more complex secondary structures.

Figure 2b − d shows a particular kind of hierarchical structures produced via the femtosecond laser ablation and the *in-situ* deposition process. The specific laser processing conditions applied were 5 W, 200 kHz, 500 mm/s, 50 μm, and 125 for the average laser power, pulse repetition rate, scanning speed, interval of scanning lines, and repeated number of laser scans, respectively. The whole structural layout was composed of arrayed micro cones separated by deep valleys which were direct results of the femtosecond laser subtractive ablation process (Fig. 2b). The side walls of these micro cones were covered by nano ripples (the outer areas of the red dash rectangle in Fig. 2c), which are also typical structural features being induced during femtosecond laser irradiation. In addition to the typical arrayed microscale structures and induced nanoscale structures, secondary structures could also be observed on the top of each micro cone (Fig. 2c). The lateral widths of the secondary structures are around 20 μm, and the secondary structures are further composed of sub-micro and nanoscale particles (Fig. 2d). Both the micro cones and nano ripples were directly and simultaneously formed by the femtosecond laser ablation, while the secondary structures were formed by the *in-situ* deposition process which occurred following the laser ablation. During continuous laser scanning from one line to another as well as the repeat of laser scanning, the subtractive process via ablation and the micro-additive process via *in-situ* deposition occurred alternately and sequentially. As a result, prominent hierarchical structures were fabricated.

**Fig. 3:**
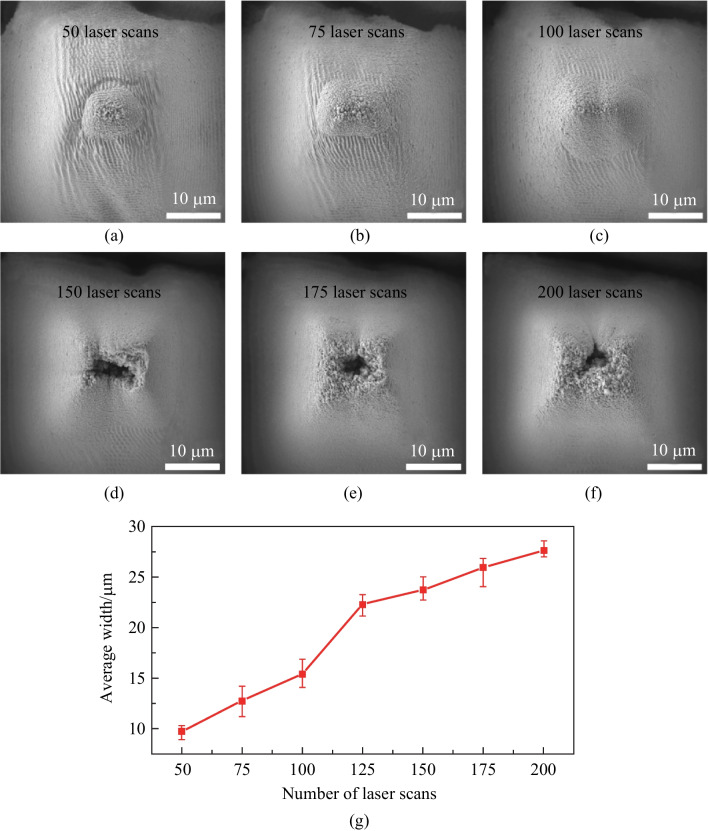
Evolution of the fort-like secondary structures on top of the primary micro cones with a periodicity of 50 μm. **a** − **f** SEM images of the hierarchical structures formed through 50, 75, 100, 150, 175, and 200 laser scans, respectively. **g** Evolution trend of the average lateral widths of the fort-like structures with the repeated number of laser scans. Error bars show the size variations of the fort-like structures measured at different positions on the same surfaces

Further, Fig. 3a − f shows that the *in-situ* deposition-based micro-additive process could be controlled by adapting the femtosecond laser processing conditions. Firstly, the repeated number of laser scans was a crucial factor for the formation of the secondary structures via the additive *in-situ* deposition process. As indicated by the evolution from Fig. 3a to Fig. 3f, the secondary structures grew larger as more laser scans were conducted. The formation of the secondary structures via the micro-additive process on top of the primary micro cones is just like building up forts on micro hills. Specifically, the appearance and evolution of the fort-like structures from Fig. 3a to Fig. 3c was clear evidence for their formation in a localized additive manner. As the laser ablation was continuously run from 50 scans (Fig. 3a) to 200 scans (Fig. 3f), the fort-like structures were continuously manifested, with their lateral width increased from below 10 μm to about 28 μm (Fig. 3g). To the best of our knowledge, such a unique kind of hierarchical surface structures with the fort-like secondary features sitting on the primary micro cone arrays have not been reported previously. Multiple-step approaches are usually needed to fabricate or attach additional structural features on the primary structures produced via ultrafast laser ablation. We succeeded in demonstrating that the *in-situ* deposition during ultrafast laser ablation could be intentionally utilized as a fabrication process and could be controlled together with the subtractive ablation process. This may inspire a deeper thinking on further exploration of the versatility of ultrafast lasers in fabricating surface micro/nano structures.

It should be noted that the micro-additive process demonstrated here was a clear departure from commonly used laser additive manufacturing techniques relying on either external material feeding (e.g., metal powders in selective laser melting) or special non-linear photochemical mechanisms (e.g., two-photon polymerization) [38, 39]. The *in-situ* deposition based micro-additive process was not an individual fabrication approach but occurred along with the typical femtosecond laser subtractive ablation process. The subtractive ablation not only fabricated the primary structures but also provided the material source (i.e., the plasma formation) for the micro-additive building up of the fort-like secondary structures. We further demonstrated that the sequential femtosecond laser subtractive and additive processes could both be controlled, to produce a hybrid fabrication approach, facilitating the production of surface structures with more hierarchy and complexity.

**Fig. 4:**
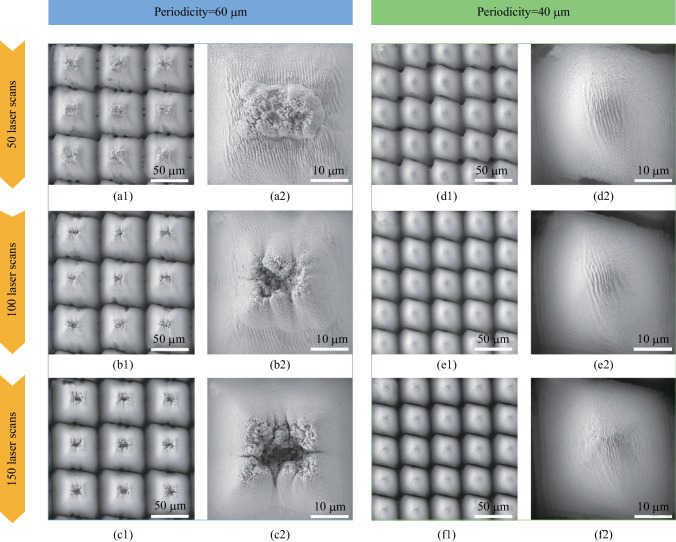
SEM images of the fort-like structures sitting on micro cone arrays with different periodicities. Parts **a1**–**c1** show micro cone arrays with a periodicity of 60 μm formed through 50, 100, and 150 laser scans, respectively. Parts **d1**–**f1** show micro cone arrays with a periodicity of 40 μm formed through 50, 100, and 150 laser scans, respectively. Parts **a2**− **f2** are magnified SEM images of (**a1** − **f1**). All laser processing conditions are the same as in Figs. 2 and 3 except the line intervals (equal to the periodicities of micro cones)

In addition to the repeated number of laser scans, the scanning patterns were also important for triggering the femtosecond laser sequential subtractive and additive fabrication process, and thus producing the specific hierarchical structures with fort-like structures sitting on arrayed micro cones. Compared to the condition with a 50 μm interval between scanning lines as shown in Figs. 2 and 3, the fort-like structures were easier to form and became more prominent when a larger scanning-line interval (e.g., 60 μm) was used, as shown in Fig. 4a1 − c2. As also shown in Fig. 3, the fort-like secondary structures grew larger when more laser scans were applied. However, for the condition with a smaller scanning-line interval (e.g., 40 μm), the fort-like (or any other) secondary structures could not be formed under the same laser power regardless of how many laser scans were used, as shown in Fig. 4d1 − f2, indicating that the localized micro-additive process failed to occur.

**Fig. 5:**
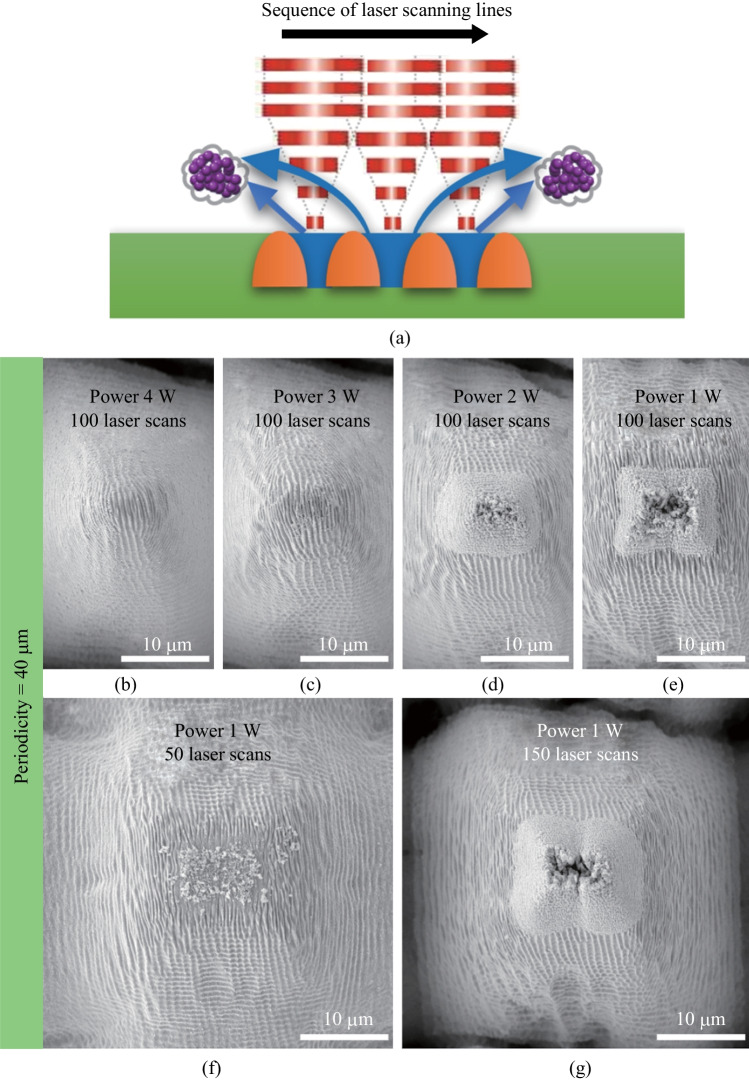
**a** Schematic illustration of femtosecond laser sequential scanning process with small scanning-line intervals. **b** − **e** SEM images for top of the micro cones with a periodicity of 40 μm formed after 100 laser scans under laser power of 4, 3, 2, and 1 W, respectively. **f**, **g** SEM images of top of micro cones formed with a laser power of 1 W after 50 and 150 laser scans, respectively

It is suggested that the surface areas between adjacent scanning lines, which were not ablated by femtosecond laser pulses, also determined the occurrence of the *in-situ* deposition based micro-additive process to form the hierarchical structures. When scanning-line intervals were larger than the diameter of laser spot, there was sufficient surface area left both for supporting the *in-situ* deposited particles and for ensuring that the *in-situ* deposited particles were not removed by the following scanning lines. Subsequently, during the repeat of laser scanning, more nanoparticles could be further deposited onto these areas, and thus the fort-like secondary structures could be constructed there gradually, through the micro-additive process. On the other hand, when scanning-line intervals were near or even smaller than the diameter of laser spot, the ablation-based subtractive and *in-situ* deposition-based additive processes impacted each other during the continuous laser scanning. As a result, the whole solid surfaces were directly ablated, leaving no supporting areas for the fort-like structures to build up, as shown in Fig. 5a. The *in-situ* deposited particles could even be re-ablated by both following scanning lines and following laser scans. Therefore, few *in-situ* deposited particles could remain on the surfaces after ending of laser ablation, yielding no fort-like secondary structures.

The above analyses were further testified by investigating the influence of laser power on the formation of the fort-like hierarchical structures. According to the intensity profile of a Gaussian beam, it is possible, by carefully tuning the average laser power, to reduce the laser intensity at the peripheral region of a laser beam down to below the material ablation threshold while keep the laser intensity at its central region still above the ablation threshold. Although the apparent diameter of the focused laser spot was not reduced, the surface areas that could be ablated by a femtosecond laser were actually reduced, which can be regarded as a process to compensate for the influence of the scanning-line intervals. As a result, surface areas for supporting the *in-situ* deposited particles are expected to be retained in the case where scanning-line intervals are smaller, and consequently the fort-like structures can be piled up via the localized micro-additive process.

As a verification, lower laser power was tested during femtosecond laser ablation with the scanning-line interval of 40 μm. As can be seen in Fig. 5b − e, when laser power gradually decreased from 4 to 1 W, the fort-like secondary structures did not appear at first (4 and 3 W), and then showed up obviously after the laser power fell to a certain value (2 and 1 W), although both the repeated number of laser scans and the scanning-line intervals were kept constant. Therefore, the effectiveness of using laser power to compensate the influence of scanning-line intervals was verified, showing the flexibility in controlling the *in-situ* deposition based micro-additive process. Moreover, it was noticed that the difference between the conditions of 2 W (Fig. 5d) and 1 W (Fig. 5e) was less obvious than that between the conditions of 3 W (Fig. 5c) and 2 W (Fig. 5d). It indicates that using lower laser power could help to preserve supporting areas for the micro-additive process but at a cost of reduced amount of the plasma formation and subsequent *in-situ* deposition. As a balanced result, the fort-like structure formed using 1 W laser power (Fig. 5e) was not obviously larger than that using 2 W laser power (Fig. 5d).

When a fixed laser power was used, the repeated number of laser scans could exert a more obvious effect in controlling the localized micro-additive process, thus controlling the sizes of the fort-like structures. Under the lower laser power and smaller scanning-line interval condition (i.e., 1 W and 40 μm), the growing trend of the fort-like structures with the number of laser scans (Fig. 5e − g) was consistent with that observed under the higher larger and larger scanning-line interval condition (i.e., 5 W and 50 μm, Fig. 3).

The investigations above on using the “laser power—line intervals—laser scans” parameter combinations to finely control the femtosecond laser sequential subtractive and additive processes provide a clue that there is always more to explore within the ultrafast laser-matter interactions, based on which novel micro/nanofabrication approaches can be possibly developed. It is worth noting that this study is not specialized for a particular application. However, the fabrication of surface micro/nano structures with controlled dimensions, hierarchies, and compositions is a capability commonly required for realizing various surface functions. The demonstrations in this research may inspire future structure design and fabrication for achieving desired surface properties.

## Conclusions

In summary, we demonstrated in this study that the femtosecond laser irradiation on solid surfaces could enable not only the common subtractive ablation process but also a unique localized micro-additive process based on the *in-situ* deposition of plasma plumes. A systematic investigation was conducted to clarify the influences of the “laser power—line intervals—laser scans” parameter sets, with the critical femtosecond laser processing conditions for both processes to occur sequentially being established. Through the subtractive and micro-additive hybrid approach, a particular kind of hierarchical structure, featuring fort-like structures sitting on arrayed micro cones, was fabricated in a controllable manner on tungsten surface. It was observed that the *in-situ* deposition within ultrafast laser processing can be utilized as a fabrication process to pile up more complex hierarchical structures. The uncovered ultrafast laser-matter interaction mechanisms and the efforts to gain more control over the ultrafast laser micro/nanofabrication approaches can motivate future research interests to explore new possibilities in the fabrication of functional surface micro/nano structures using ultrafast lasers.

## Data Availability

The data that support the findings of this study are available from the corresponding author, upon reasonable request.
